# Assembly and Comparative Analysis of the Complete Mitochondrial Genomes of *Smilax glabra* and *Smilax zeylanica*

**DOI:** 10.3390/genes16040450

**Published:** 2025-04-14

**Authors:** Guojian Liao, Wenjing Liang, Haixia Yu, Kun Zhang, Linxuan Li, Shixin Feng, Lisha Song, Cuihong Yang, Lingyun Wan, Dongqiang Zeng, Zhanjiang Zhang, Shugen Wei

**Affiliations:** 1 Guangxi Key Laboratory of High-Quality Formation and Utilization of Dao-Di Herbs, Guangxi Botanical Garden of Medicinal Plants, Nanning 530023, China; liaoguojian1990@outlook.com (G.L.); l915681559@163.com (W.L.); yuhaixia0201@163.com (H.Y.); shixin1996@126.com (S.F.); lishasong@126.com (L.S.); yangcuihong3@163.com (C.Y.); wanlingyun1984@163.com (L.W.); 2 College of Agriculture, Guangxi University, Nanning 530024, China; zengdq550@163.com; 3 Guangxi Traditional Chinese Medicine Breeding Technology Innovation Center, Guangxi Botanical Garden of Medicinal Plants, Nanning 530023, China; zhkun_lc@126.com; 4 National Center for Traditional Chinese Medicine Inheritance and Innovation, Guangxi Botanical Garden of Medicinal Plants, Nanning 530023, China; lilinxuan1125@163.com; 5 National Engineering Research Center for the Development of Southwestern Endangered Medicinal Materials, Guangxi Botanical Garden of Medicinal Plants, Nanning 530023, China

**Keywords:** *Smilax glabra*, *Smilax zeylanica*, mitochondrial genome, RNA editing, de novo assembly, phylogenetic analysis

## Abstract

Background: *Smilax glabra* (*S. glabra*) and *Smilax zeylanica* (*S. zeylanica*), two medicinally important species within the genus *Smilax*, have been widely used in Traditional Chinese Medicine (TCM) for the treatment of rheumatism, traumatic injuries, and related ailments. Despite their medicinal significance, research on the mitochondrial DNA (mtDNA) of *Smilax* species remains limited. Methods: We utilized NovaSeq 6000 and PromethION sequencing platforms to assemble the complete mitochondrial genomes of *Smilax glabra* and *Smilax zeylanica*, and conducted in-depth comparative genomic and evolutionary analyses. Results: The complete mitochondrial genomes of *S. glabra* and *S. zeylanica* were assembled and annotated, with total lengths of 535,215 bp and 471,049 bp, respectively. Both genomes encode 40 unique protein-coding genes (PCGs), composed of 24 core and 16 non-core genes, alongside multiple tRNA and rRNA genes. Repetitive element analysis identified 158 and 403 dispersed repeats in *S. glabra* and *S. zeylanica*, respectively, as well as 123 and 139 simple sequence repeats (SSRs). RNA editing site predictions revealed C-to-U conversions in both species. Additionally, chloroplast-to-mitochondrial DNA migration analysis detected 34 homologous fragments in *S. glabra* and 28 homologous fragments in *S. zeylanica*. Phylogenetically, *S*. *glabra* and *S. zeylanica* cluster within the Liliales order and Smilacaceae family, closely related to Lilium species. Collinearity analysis indicated numerous syntenic blocks between Smilax and three other Liliopsida species, though gene order was not conserved. Conclusions: This study presents high-quality mitochondrial genome assemblies for *S*. *glabra* and *S. zeylanica*, providing valuable insights into molecular identification and conservation efforts of these traditional medicinal plants.

## 1. Introduction

The genus *Smilax*, part of the family *Liliaceae*, includes around 300 species distributed worldwide, primarily in tropical and subtropical regions, with the greatest diversity observed in the Americas and Asia [1]. In China, more than 60 species of *Smilax* are widely distributed, particularly in the regions south of the Yangtze River. These plants have garnered significant attention due to their medicinal value and have been extensively used in TCM for millennia. According to the Pharmacopoeia of the People’s Republic of China (2020 edition), several species within the genus *Smilax* are of notable medicinal importance, including *S. glabra* and *S. zeylanica* [2,3,4].

*S. glabra* and *S. zeylanica* are two medicinal plants of significant therapeutic value. The rhizomes of *S. glabra*, widely used in TCM, are known for their effects in eliminating dampness, detoxifying, clearing heat, and dispelling wind-dampness [5]. They are commonly employed to treat conditions such as damp-heat strangury, rheumatic arthralgia, and cancer [6]. Modern pharmacological studies have revealed that *S. glabra* contains various bioactive compounds, including polysaccharides, saponins, and flavonoids, which exhibit anti-inflammatory, antioxidant, and immunomodulatory properties. On the other hand, *S. zeylanica* is recognized for its ability to clear heat, detoxify, dispel wind-dampness, and promote blood circulation to remove stasis [7]. The primary active components of *S. zeylanica* include saponins and flavonoids, which contribute to its anti-inflammatory, analgesic, and antitumor pharmacological effects [8]. Although both *S. glabra* and *S. zeylanica* are extensively utilized in TCM, they differ significantly in their origins, chemical compositions, and primary therapeutic effects. *S. glabra* is more focused on detoxification and dampness elimination, whereas *S. zeylanica* demonstrates superior efficacy in anti-inflammatory and analgesic applications [9]. Additionally, due to their morphological similarities, these two species are often misused. Therefore, accurate identification and authentication of these medicinal materials are crucial to ensuring the efficacy, consistency, and safety of TCM. In recent years, advancements in molecular techniques, such as DNA barcoding, have been employed to identify herbal medicines, providing a scientific basis for species discrimination in TCM [10]. Previous work has utilized mitochondrial genes such as *nad1* (regions b, c) and *nad5* (regions d, e) for species-level identification, e.g., in *Gentiana macrophylla* [11].

Mitochondria serve as critical intracellular organelles that significantly influence plant growth, developmental progression, and adaptation to environmental stresses [12]. As the key central for cellular energy metabolism, they synthesize ATP through oxidative phosphorylation to fuel cellular activities. The mitochondrial genome (mtDNA) forms the genetic foundation of mitochondrial function, exhibiting remarkable complexity and diversity in its structure and organization across plant species [13]. Plant mtDNA is generally large, ranging from 200 to 2500 kb, significantly larger than that of animals. This complexity primarily arises from the abundance of repetitive sequences within plant mitochondrial genomes, which, through homologous recombination mechanisms, contribute to the diversity of genomic structures, including circular, linear, or multi-branched configurations [14]. These characteristics of the mitochondrial genome make it invaluable for studies on species evolution. Its highly conserved sequences and multi-copy nature render it an ideal tool for species identification and phylogenetic analysis. By comparing the sequence features of mtDNA among different species, insights into the genetic relationships and evolutionary history among species can be revealed [15]. Furthermore, evolutionary analysis of mtDNA offers important insights into the dynamic changes in genome structure. In plants, research on mitochondrial genomes not only aids in comprehending the evolutionary trajectory of plants but also offers theoretical support for genetic improvement and resource conservation [16]. For instance, analyzing RNA editing events and repetitive sequences in mitochondrial genomes can enhance our understanding of the evolutionary mechanisms underlying plant genomes [17]. Additionally, the diversity of mitochondrial genomes provides potential genetic resources for plant adaptation to environmental changes [18]. *S. zeylanica* and *S. glabra*, which belong to the genus *Smilax* in the family *Liliaceae*, are important contributors to TCM. However, despite their widespread use in traditional medicine, research on the mitochondrial genomes of these two plants remains in its infancy. Previous studies have primarily focused on their pharmacologically active components and traditional applications, with limited exploration into the identification, lineage studies, and phylogenetic analysis of their mitochondrial genomes. To date, the mitochondrial genomes of Smilax species have not been sequenced. Therefore, there is an urgent need for in-depth research into the genomic characteristics of *S. zeylanica* and *S. glabra*, utilizing their complete mitochondrial genomes for comparative and evolutionary analysis.

Given the significance of mitochondrial genomes in plant evolution and phylogenetic analysis, this study focuses on the mitochondrial genomes of two important medicinal plants from the genus *Smilax* in the family *Liliaceae: S. zeylanica* and *S. glabra*. Utilizing NovaSeq 6000 and PromethION sequencing technologies, we obtained the complete mitochondrial genome sequences of these two plants. Our objective was to conduct a comprehensive analysis of their genomic characteristics and genetic mechanisms, thereby establishing a theoretical basis for the development of molecular markers. Through a multifaceted structural analysis, we systematically evaluated the fundamental features of the mitochondrial genomes of *S. zeylanica* and *S. glabra*, including gene composition, codon usage bias, distribution of repetitive sequences, GC content, and RNA editing events. Additionally, we retrieved mitochondrial genomes of other related species from the NCBI database and employed comprehensive collinearity mapping to compare the genomic synteny of *S. zeylanica* and *S. glabra* with those of other species, unveiling gene recombination and rearrangement events during their evolutionary processes. Moreover, we analyzed homologous sequences between chloroplast and mitochondrial genomes, followed by phylogenetic reconstruction through maximum likelihood analysis of PCGs across 20 plant species. This study not only offers new insights into the evolutionary trajectory of Smilax species but also provides crucial scientific evidence for the molecular identification and conservation of traditional medicinal resources. It establishes a solid foundation for future research on plant molecular diversity and evolution.

## 2. Materials and Methods

### 2.1. DNA Extraction of Sample and Mitogenomic Sequencing

The leaves of plants were frozen in liquid nitrogen and stored at −80 °C. Total genomic DNA of *S. zeylanica* and *S. glabra* was extracted using the cetyltrimethyl ammonium bromide (CTAB) method. The purity and concentration of extracted DNA were assessed using both a NanoDrop One spectrophotometer (Thermo Fisher Scientific, Wilmington, DE, USA) and a Qubit 3.0 Fluorometer with dsDNA HS Assay Kit (Thermo Fisher Scientific, Carlsbad, CA, USA). Subsequently, the qualified and quantified sample libraries were sequenced via NovaSeq 6000 (Illumina, San Diego, CA, USA) and PromethION (Oxford Nanopore Technologies, Oxford, UK) platforms. Quality control of raw sequencing data was performed. We performed stringent quality control on the raw data generated from Illumina sequencing, following these specific steps: (1) removal of adapter sequences from the reads; (2) deletion of bases at the 5′ end that contained non-AGCT nucleotides; (3) trimming the ends of low-quality reads with a quality value < Q20; (4) removal of reads containing more than 10% of N. Additionally, for the ONT PromethION platform, the quality control standard involved the removal of sequences with an average quality value less than or equal to 7.

### 2.2. Assembly and Annotation of the Mitogenome

First, the mitochondrial genomes of *S. zeylanica* and *S. glabra* were assembled based on long-read sequencing data. The Flye software (v2.8.3) [19] was used with default settings to directly assemble the long-read data, producing a graphical assembly in GFA format. A local database was created for all assembled contigs in FASTA format using makeblastdb. The BLASTn tool was subsequently used to find contig fragments with mitochondrial genes, utilizing the mitochondrial genes of *Arabidopsis (Arabidopsis thaliana)* as reference sequences. The BLASTn parameters were set as follows: -evalue 1 × 10^−5^-outfmt 6-max_hsps 10-word_size 7-task blastn-short. The GFA file was visualized using Bandage software (v0.8.1) [20], and mitochondrial contigs were selected based on the BLASTn results, yielding a draft mitochondrial genome for the species. Next, the alignment of the long-read and short-read data to the mitochondrial contigs was performed using BWA software (v0.7.17) [21]. Reads aligned to the mitochondrial contigs were filtered and extracted for subsequent hybrid assembly. Finally, a hybrid assembly strategy was employed to assemble the mitochondrial genome of *S. zeylanica* by integrating both short-read and long-read sequencing data. Unicycler [22] was used with default parameters to perform the hybrid assembly, resulting in the final mitochondrial genome of *S. zeylanica*. The Bandage software (v0.8.1) was employed for the assembled mitochondrial genome visualization. The Geseq software (v2.03) was used to annotate PCGs in the mitochondrial genome, using the reference genomes of *Arabidopsis* (NC_037304) and *Liriodendron tulipifera* (NC_021152.1) [23]. Additionally, the mitochondrial genome was annotated using the IPMGA tool (http://www.1kmpg.cn/ipmga/, accessed on 8 August 2024), which is particularly effective for annotating splice sites and trans-splicing genes in angiosperm mitochondrial genomes. The annotation of transfer RNAs (tRNAs) was performed with tRNAscan-SE software (v2.0.11) [24], while ribosomal RNAs (rRNAs) were annotated using BLASTN software (v2.13.0) [25]. Any mistakes in the mitochondrial genome annotations were manually fixed using Apollo software (v1.11.8) [26].

### 2.3. Analysis of Repeat Fragments, Codon Usage Bias, and Prediction of RNA Editing Sites

The PCGs of the mitochondrial genome were extracted using Phylosuite software (v1.1.16) [27]. Codon usage bias analysis of the mitochondrial PCGs was performed using MEGA software (v7.0) [28], and the Relative Synonymous Codon Usage (RSCU) values were calculated. Repetitive sequences, including microsatellites, tandem repeats, and dispersed repeats, were identified by MISA (v2.1) [29], TRF (v4.09) [30], and the REPuter web server [31], respectively. The data visualization was performed using Excel (2021) and the Circos package (v0.69.9) [32]. All PCGs encoded by the mitochondrial genome of the species were used as input files for predicting C-to-U RNA editing sites in mitochondrial PCGs using Deepred-mt [33]. This tool employs a Convolutional Neural Network (CNN) model for prediction, demonstrating higher accuracy compared to previous prediction tools. All results with a probability value greater than 0.9 were retained.

### 2.4. Sequence Transfer Analysis

The chloroplast genome was assembled utilizing the GetOrganelle (v1.6.4) pipeline for sequence transfer analysis [34]. CPGAVAS2 was utilized to annotate the assembled chloroplast genome [35], followed by manual refinement of annotations using the CPGView software 2023 [36]. BLASTN (v2.13.0) [25] was employed to identify homologous segments, and Circos (v0.69.9) [32] was used to visualize the alignment results.

### 2.5. Construction of Maximum Likelihood Tree Based on the PCGs

We retrieved 19 complete mitochondrial genome sequences from the NCBI database. These sequences represent five different orders: Asparagales, Liliales, Arecales, Ranunculales, and Alismatales. We utilized PhyloSuite software (v1.1.16) to extract orthologous gene sequences [27]. The MAFFT algorithm (v7.505) was employed to conduct multiple sequence alignments [37]. A phylogenetic tree was created using the maximum likelihood (ML) approach with IQ-TREE software (v1.6.12) [38], configured with “--alrt 1000-B 1000”. The phylogenetic tree was visualized employing the Interactive Tree of Life (ITOL) platform (version 6) [39].

### 2.6. Identification of Homologous Fragments and Collinear Analysis

Conserved homologous sequences between *Smilax* and the *Liliales* were identified by the BLASTn program with the following parameters: ‘-word_size 9, -evalue 1 × 10^−5^, -gapopen 5, -reward 2, -penalty-3,-gapextend 2’. Only collinear blocks exceeding 500 bp in length were selected for further analysis. MCscanX (v1.5.2) [40] was employed to generate multiple synteny plots from BLASTn-based pairwise alignments, revealing conserved syntenic regions across genomes.

## 3. Results

### 3.1. Genomic Characteristics of the Smilax Mitochondrial Genomes

**Figure 1:**
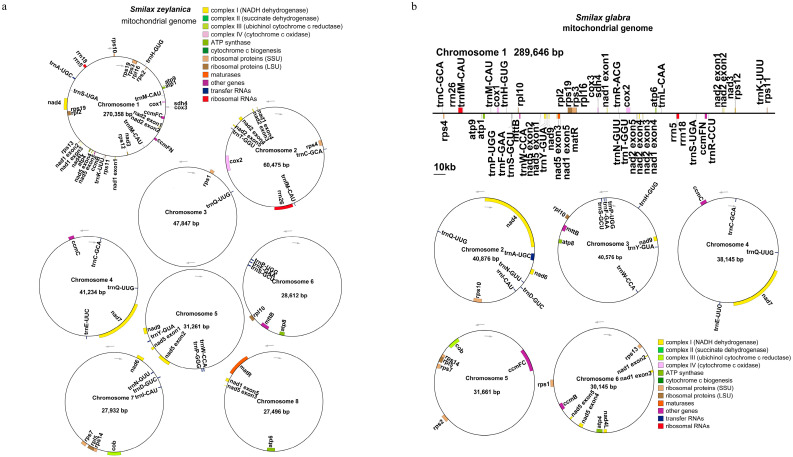
Mitochondrial genome map of *S. zeylanica* (**a**) and *S. glabra* (**b**). Circular representation of the mitochondrial genome of S. *zeylanica* (**a**) and *S. glabra* (**b**), depicting gene organization, coding regions, and non-coding regions. Different colors indicate distinct gene categories. Genes (exons are shown as closed boxes) shown outside the curve are transcribed counterclockwise, whereas those inside are transcribed clockwise.

We assembled and annotated the mitochondrial genomes of *S. zeylanica* and *S. glabra*. The primary structure of the *S. zeylanica* mitochondrial genome exhibits a multi-branched configuration. After excluding repetitive regions using Nanopore sequencing data, a total of eight circular contigs were obtained, with a combined length of 535,215 bp and a GC content of 46.05% (Figure 1a). Similarly, the *S. glabra* mitochondrial genome also displays a multi-branched structure. Following the same processing method, one linear contig and five circular contigs were obtained, totaling 471,049 bp in length with a GC content of 46.16% (Figure 1b).

In terms of gene annotation, both the *S. zeylanica* and *S. glabra* mitochondrial genomes were annotated with 40 unique PCGs, including 24 core mitochondrial genes and 16 non-core genes (Table S1). Additionally, the *S. zeylanica* mitochondrial genome contains 19 tRNA genes (3 of which are multi-copy) and 3 rRNA genes, while the *S. glabra* mitochondrial genome comprises 21 tRNA genes (9 of which are multi-copy) and 3 rRNA genes. The core genes are highly conserved in both genomes, including nine NADH dehydrogenase genes (*nad1*–*nad9*), five ATP synthase genes (*atp1*, *atp4*, *atp6*, *atp8*, and *atp9*), three cytochrome C oxidase genes (*cox1*–*cox3*), four cytochrome C biogenesis genes (*ccmB*, *ccmC*, *ccmFC*, and *ccmFN*), one membrane transport protein gene (*mttB*), one maturase gene (*matR*), and one ubiquinol-cytochrome C reductase gene (*cob*). The non-core genes include four ribosomal large subunit genes (*rpl2*, *rpl5*, *rpl10*, *and rpl16*), eleven ribosomal small subunit genes (*rps1*–*rps4*, *rps7*, *rps10*–*rps14*, and *rps19*), and one succinate dehydrogenase gene (*sdh4*). The mitochondrial genomes of *S. zeylanica* and *S. glabra* exhibit high similarity in both structure and gene composition, although differences exist in the number and copy count of tRNA genes.

### 3.2. Analysis of Relative Synonymous Codon Usage

**Figure 2:**
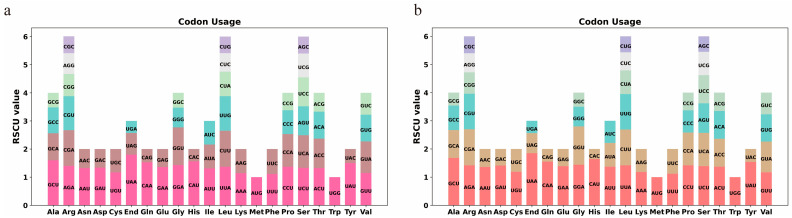
(**a**) *S. zeylanica* and (**b**) *S. glabra* mitogenome relative synonymous codon usage (RSCU). The *x*-axis represents codon families and the *y*-axis displays the RSCU values, which quantify the ratio between the observed frequency of a specific codon and its expected frequency under the assumption of uniform synonymous codon usage. Colors denote codon types for each amino acid.

A codon usage bias analysis was performed on 40 distinct protein-coding genes from the mitochondrial genomes of *S. glabra* and *S. zeylanica*, and the results are summarized in Table S2. Statistical analysis of codon usage patterns demonstrated that mitochondrial PCGs in both species exhibited preferential usage of specific codons (RSCU > 1). Notably, only the start codon AUG and tryptophan (UGG) showed neutral usage (RSCU = 1), while all other amino acids displayed biased codon selection (Figure 2a). In the mitochondrial genome of *S. glabra*, the stop codon UAA exhibited the highest RSCU value of 1.85, indicating a strong usage preference, followed by the alanine (Ala) codon GCU, with an RSCU value of 1.68. Similarly, in the mitochondrial genome of *S. zeylanica*, the stop codon UAA had the highest RSCU value of 1.80, and the alanine (Ala) codon GCU was next, with an RSCU value of 1.60 (Figure 2b). These findings suggest that the mitochondrial PCGs of *S. glabra* and *S. zeylanica* exhibit similar patterns of codon usage bias, particularly in the usage of stop codons and alanine codons.

### 3.3. Repeat Sequence Analysis

**Figure 3:**
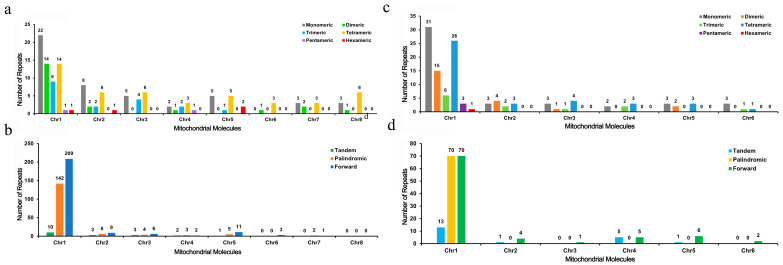
Histogram of Repeat Sequence Analysis. (**a**,**c**). The *x*-axis represents mitochondrial molecules, and the *y*-axis represents the number of repeat fragments. The gray legend denotes monomer SSRs, the orange legend denotes dimer SSRs, the green legend denotes trimer SSRs, the blue legend denotes tetramer SSRs, the purple legend denotes pentamer SSRs, and the red legend denotes hexamer SSRs. (**b**,**d**). The *x*-axis represents mitochondrial molecules, and the *y*-axis represents the number of repeat fragments. The blue legend indicates tandem repeats, the yellow legend indicates palindromic repeats, and the green legend indicates forward repeats.

In the mitochondrial genomes of *S. glabra* and *S. zeylanica*, 158 and 403 dispersed repeat sequences (≥30 bp) were identified, respectively. Among these, *S. glabra* contained 88 forward repeats and 70 palindromic repeats, whereas *S. zeylanica* exhibited 240 forward repeats and 163 palindromic repeats (Figure 3b,d). Additionally, 123 and 139 SSRs were identified in the mitochondrial genomes of *S. glabra* and *S. zeylanica*, respectively. Figure 3a,c illustrates the proportion of each repeat type. In *S. glabra*, dimeric repeats accounted for 36.6% of the total, followed by tetrameric repeats at 32.5%. In *S. zeylanica*, monomeric repeats constituted 36.7% of the total, followed by tetrameric repeats at 34.5%.

### 3.4. RNA Editing Event Prediction

**Figure 4:**
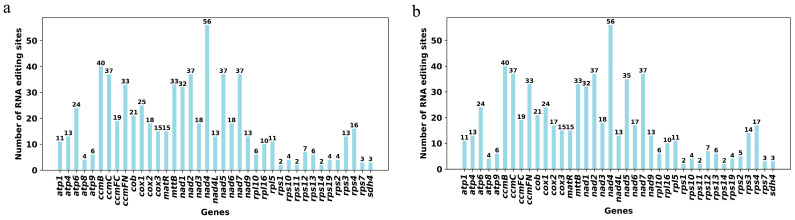
The number of predicted RNA editing sites in each PCG of the mitochondrion. (**a**) *S. zeylanica*; (**b**) *S. glabra*. The *x*-axis represents gene names, the *y*-axis indicates the number of RNA editing sites.

We identified RNA editing events in the 40 unique PCGs of the mitochondrial genomes of *S. zeylanica* and *S. glabra*, using a cutoff value of 0.9 for detection. Under this criterion, 668 potential RNA editing sites were identified in the 40 PCGs of the *S. zeylanica* mitochondrial genome, with all editing events involving C-to-U base conversions (Figure 4a). Similarly, 666 potential RNA editing sites were identified in the 40 PCGs of the *S. glabra* mitochondrial genome, also exclusively involving C-to-U conversions (Figure 4b). In both mitochondrial genomes, the *nad4* gene exhibited the highest number of RNA editing sites, totaling 56, followed by the *ccmB* gene, with 40 RNA editing sites identified in each genome. These findings indicate a high degree of similarity in the distribution and type of RNA editing events in the mitochondrial genomes of *S. zeylanica* and *S. glabra*, with the *nad4* and *ccmB* genes likely serving as major targets for RNA editing.

### 3.5. DNA Transfer from Chloroplast to Mitochondria

**Figure 5:**
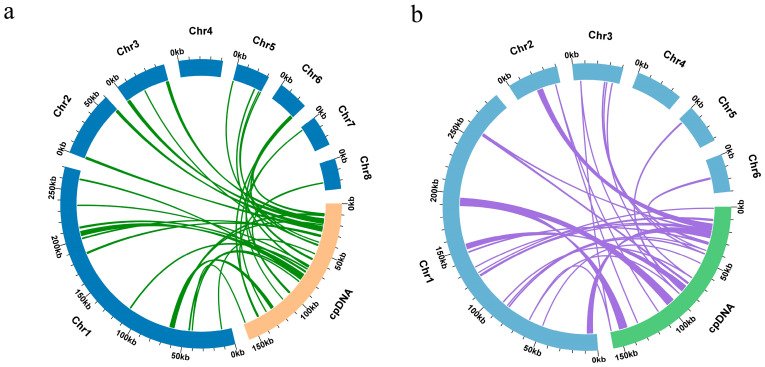
Sequence migration analysis. (**a**) *S. zeylanica*; (**b**) *S. glabra*. The mitochondrial genome is shown by blue arcs, the chloroplast genome by yellow/green arcs, and the green/purple lines that connect the arcs correspond to homologous genome fragments.

During the evolution of higher plants, the movement of genetic material within cells frequently occurs in mitochondrial genomes. However, it is noteworthy that these sequences derived from chloroplast organelles tend to have a relatively low retention rate. Therefore, we analyzed the DNA transfer from the chloroplast to the mitochondrial organelles in *S. zeylanica* and *S. glabra*. In the species *S. zeylanica*, a total of 34 homologous segments between mitochondrial and chloroplast genomes were identified, with a cumulative length of 24,711 bp, accounting for 4.62% of the total mitochondrial genome length. The longest homologous segment was MTPT21, with a length of 4169 bp. Annotation of these homologous segments revealed the presence of nine complete genes, including six protein-coding genes (*ndhH*, *petD*, *rpl14*, *rpoA*, *rps2*, *rps11*), and three tRNA genes (*trnC*-*GCA*, *trnH*-*GUG*, *trnN*-*GUU*) (Figure 5b). In the species *S. glabra*, a total of 28 homologous segments between mitochondrial and chloroplast genomes were identified, with a cumulative length of 26,997 bp, accounting for 5.73% of the total mitochondrial genome length. The longest homologous segment was MTPT6, with a length of 6813 bp. Annotation results showed that these homologous segments contained twelve complete genes, including six protein-coding genes (*ndhB*, *petD*, *rpl14*, *rpoA*, *rps7*, *rps11*) and six tRNA genes (*trnC*-*GCA*, *trnH*-*GUG*, *trnL*-*CAA*, *trnM*-*CAU*, *trnN*-*GUU*, *trnW*-*CCA*) (Figure 5b). These findings highlight the occurrence of chloroplast-to-mitochondria DNA transfer in *S. zeylanica* and *S. glabra* and underscore the presence of several complete genes within these transferred segments.

### 3.6. Phylogenetic Analysis Within the Mitochondrial Genome

**Figure 6:**
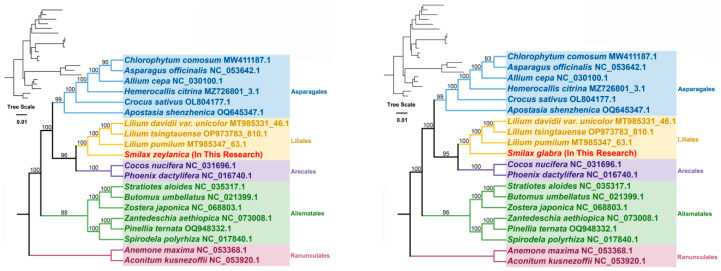
Phylogenetic analysis. Phylogenetic tree of 20 angiosperms based on the sequences of 24 conserved mitochondrial PCGs. The number at each node is the bootstrap probability. The different colors represent five distinct orders: *Asparagales*, *Liliales*, *Arecales*, *Alismatales*, and *Ranunculales*.

A phylogenetic tree was created using DNA sequences from 24 conserved mitochondrial PCGs across 20 species from five angiosperm orders to clarify the evolutionary path of the *Smilax* mitochondrial genome (Figure 6). The specific mitochondrial genome sequences of the plant species are listed in Supplementary Materials Table S1. The shared PCGs include *atp1*, *atp4*, *atp6*, *atp8*, *atp9*, *ccmB*, *ccmC*, *ccmFC*, *ccmFN*, *cob*, *cox1*, co*x*2, *cox3*, *matR*, *mttB*, *nad1*, *nad2*, *nad3*, *nad4*, *nad4L*, nad5, *nad6*, *nad7*, and *nad9*. Two mitochondrial genomes from the *Ranunculales* order were designated as outgroups. According to mitochondrial DNA, the phylogenetic arrangement matches the latest classification provided by the Angiosperm Phylogeny Group (APG). *S. zeylanica* is classified under the *Liliales* order, *Smilacaceae* family, and clusters with species of the *Lilium* genus.

### 3.7. Comparative Analysis of Five Liliales Mitochondrial Genomes

**Figure 7:**
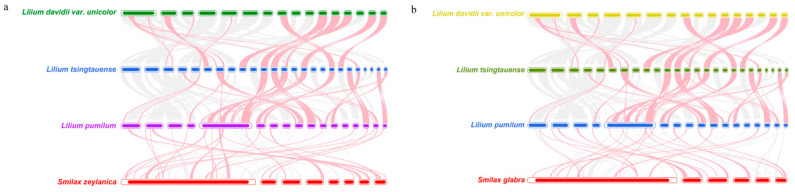
Collinearity analysis of *S. zeylanica* (**a**) and *S. glabra* (**b**). Linear bars represent mitochondrial genomes, with interconnecting ribbons denoting homologous sequences between adjacent species. The red arcuate regions indicate inverted sequences, and the gray areas represent regions with high sequence homology. The Blank regions represent sequences unique to *S. zeylanica* and *S. glabra*, showing no detectable homology with other examined species.

Mitochondrial genomes of different plant species show significant variations in structure, gene content, and gene order. The distribution of homologous sequences among species, known as homology, is often used to understand evolutionary relationships. To assess the homology of the mitochondrial genomes of Smilax plants, this study selected one representative species from each family in the phylogenetic tree for synteny analysis. The BLASTN program was employed to compare homologous genes and their sequence arrangements between Smilax and other Liliales species. Conserved syntenic blocks longer than 500 bp were identified for analysis. To better illustrate the alignment results, syntenic blocks shorter than 0.5 kb were excluded from the final results. The results revealed that Smilax and the other three Liliales species share numerous homologous syntenic blocks, although these blocks are relatively short in length. Additionally, some blank regions were identified, which are unique to the *S. zeylanica* and *S. glabra* species and show no homology with the remaining species (Figure 7a,b). The results show that the sequence of syntenic blocks in the mitochondrial genomes of these four species varies. The mitochondrial genomes of the *S. zeylanica* and *S. glabra* species have undergone genomic rearrangements compared to their closely related species, suggesting that the gene order in the mitochondrial genomes of these four Liliales species is highly non-conserved

## 4. Discussion

In this study, we sequenced, assembled, and reported the complete mitochondrial genome structures of two important medicinal plants, *S. zeylanica* and *S. glabra*. Similarly to most higher plants, the mitochondrial genomes of *S. zeylanica* and *S. glabra* exhibit complex, multi-branched structures, primarily due to the presence of numerous repeat sequences. These repeats commonly promote gene recombination and the formation of subgenomic molecules in plant mitochondrial genomes [41]. Recent studies have indicated significant structural variations in mitochondrial genomes across different plant species. For instance, the mitochondrial genomes of *Arabidopsis* and *Oryza sativa* show notable structural differences, with variations in the distribution and quantity of repeat sequences significantly impacting genome stability [42,43]. In our study, the mitochondrial genome of *S. zeylanica*, sequenced using Nanopore data after excluding repeat regions, comprised eight circular contigs with a total length of 535,215 bp and a GC content of 46.05%. In contrast, the mitochondrial genome of *S. glabra* consisted of one linear and five circular contigs, with a total length of 471,049 bp and a GC content of 46.16%. Additionally, both *S. zeylanica* and *S. glabra* mitochondrial genomes were annotated to include 40 unique protein-coding genes, containing 24 core mitochondrial genes and 16 non-core genes. These findings not only enhance our understanding of the mitochondrial genome structure of medicinal plants but also provide essential baseline data for subsequent research on the roles of the mitochondrial genome in plant evolution, metabolic regulation, and the biosynthesis of medicinal compounds.

We conducted a systematic analysis of chloroplast-derived sequences (MTPTs) in the mitochondrial genomes of *S. glabra* and *S. zeylanica*, revealing significant characteristics of interorganellar gene transfer. In the mitochondrial genome of *S. zeylanica*, a total of 34 MTPTs were identified, with an aggregate length of 24,711 bp, constituting 4.62% of the total genome length. In contrast, 28 MTPTs were identified in the *S. glabra* mitochondrial genome, with a total length of 26,997 bp, accounting for 5.73% of the total genome length. The lengths and numbers of these MTPTs are consistent with previous findings in species such as Daucus carota and Asclepias syriaca, further supporting the ubiquity of chloroplast-to-mitochondrial genome transfer [44,45]. Notably, the longest MTPTs in *S. zeylanica* and *S. glabra* were 4169 bp (MTPT21) and 6813 bp (MTPT6), respectively, indicating that both species have a significant capacity for capturing and retaining large segments of chloroplast sequences within their mitochondrial genomes. Moreover, the MTPTs in *S. zeylanica* and *S. glabra* included 9 and 12 complete genes, respectively, featuring a high retention rate of protein-coding genes (such as *ndhH*, *petD*, *rpl14*) and tRNA genes (*trnC*-*GCA*, *trnH*-*GUG*). This suggests that these genes may contribute significantly to mitochondrial genome function. For instance, the *ndhH* and *petD* genes are involved in the assembly of the NADH dehydrogenase complex and the cytochrome b6f complex, respectively, and their transfer could potentially optimize mitochondrial energy metabolism efficiency [46]. Additionally, the frequent transfer of tRNA genes (*trnC*-*GCA* and *trnH*-*GUG*) may compensate for the shortage of tRNA genes in the mitochondrial genome, thereby supporting the mitochondrial translation system [47]. These findings are consistent with the observed general pattern of chloroplast gene transfer to mitochondria in angiosperms and indicate that tRNA gene transfer is more frequent in angiosperms compared to bryophytes and gymnosperms [48]. This difference may reflect different strategies employed by various plant groups during evolution for integrating organellar genome functions.

Repeat sequences are indeed widespread in plant mitochondrial genomes and play a crucial role in intermolecular recombination within mitochondria. Previous studies have indicated that the abundance of dispersed repeat sequences reflects varying frequencies of genome recombination and evolutionary pressures [49,50]. In this study, the mitochondrial genome of *S. zeylanica* exhibited a higher number of repeat sequences. Among these, a particularly noteworthy type is microsatellites, or SSRs. SSRs are composed of short DNA fragments consisting of repeating units, typically ranging from one to six nucleotides in length [51]. SSRs have various functions and are widely used in species identification, quantitative trait locus (QTL) mapping, marker-assisted breeding, diversity analysis, and establishing evolutionary relationships. In the mitochondrial genome of *S. glabra*, a total of 123 SSRs were identified, with dimeric repeats constituting 36.6% (45 SSRs) and tetrameric repeats accounting for 32.5% (40 SSRs). In contrast, the mitochondrial genome of *S. zeylanica* contained 139 SSRs, with monomeric repeats being predominant (36.7%, 51 SSRs), followed by tetrameric repeats (34.5%, 48 SSRs). This finding is consistent with the common characteristics of SSRs in plant mitochondrial genomes, where monomeric and dimeric repeats typically have a higher proportion, although the distribution of SSR types can be influenced by the AT preference of the genome [52]. Notably, the high frequency of AT bases in SSR repeating units (such as monomeric A/T repeats and dimeric AT/TA repeats) may be associated with their susceptibility to double-strand breaks, thus promoting repeat-mediated recombination events [53]. The widespread presence of palindromic repeats (44.3% in *S. glabra* and 40.4% in *S. zeylanica*) further suggests their potential involvement in forming stable secondary structures, which could influence mitochondrial genome replication or transcription regulation [54]. The distribution characteristics of these repeat sequences not only provide potential targets for molecular marker development but also offer important clues for understanding the mechanisms of mitochondrial genome rearrangement and evolutionary adaptation in these species.

In the 40 PCGs of the *S. zeylanica* mitochondrial genome, a total of 668 potential RNA editing sites were identified, while 666 potential RNA editing sites were found in the 40 PCGs of the *S. glabra* mitochondrial genome. All editing events involved C-to-U base conversions. This result is consistent with the RNA editing patterns observed in the mitochondrial genomes of various plants, such as *Arabidopsis* and *Oryza sativa*, where widespread C-to-U editing events have been reported, indicating the high conservation of this editing type in plant mitochondria [55,56]. Notably, the *nad4* gene had the most RNA editing sites in both the *S. zeylanica* and *S. glabra* mitochondrial genomes, with 56 sites identified in each. The ccmB gene had the second highest number of RNA editing sites, with 40 sites identified in each genome. This finding suggests that *nad4* and *ccmB* genes may be primary targets for RNA editing, and their editing events might significantly influence gene function. For example, the *nad4* gene encodes a subunit of mitochondrial respiratory chain complex I, and its RNA editing could optimize protein function by altering the amino acid sequence, thus affecting mitochondrial energy metabolism efficiency [57]. Additionally, the *ccmB* gene is involved in the biosynthesis of cytochrome c, and its editing events may regulate the function of the mitochondrial electron transport chain. These results indicate a high degree of similarity in the distribution and types of RNA editing events in the mitochondrial genomes of *S. zeylanica* and *S. glabra*, further supporting the conservation and importance of RNA editing in the regulation of mitochondrial gene expression in plants.

In this study, we constructed a phylogenetic tree based on the DNA sequences of 24 conserved mitochondrial protein-coding genes across 20 species belonging to five orders of angiosperms. *S. zeylanica* and *S. glabra* were classified under the *Liliales* order, Smilacaceae family, and clustered with species from the *Lilium* genus. This result is consistent with previous studies on the classification of the Smilax genus, indicating the high reliability of our phylogenetic analysis. Additionally, the phylogenetic analysis revealed the evolutionary relationships between Smilax species and other species within the *Liliales* order, providing important insights into the evolutionary history of the Smilax genus. Significant differences exist in the structure, gene content, and gene order of mitochondrial genomes across different plant species. To assess the homology of the mitochondrial genomes of *Smilax* plants, we selected one representative species per family from the phylogenetic tree for synteny analysis. Using the BLASTN program, we compared the homologous genes and their sequence alignments between Smilax and three other species within the *Liliales* order. We focused on conserved syntenic blocks longer than 500 bp to ensure the reliability and accuracy of our results. The analysis revealed numerous homologous syntenic blocks between *Smilax* and the other three *Liliales* species, albeit with relatively short lengths. This indicates that these species have retained a degree of genomic conservation during evolution. However, we also identified some gaps—unique sequences in the *S. zeylanica* species that did not exhibit homology with other species. This suggests that the *S. zeylanica* species has undergone specific genomic changes, which may be related to its unique biological characteristics and adaptations. Moreover, the order of the syntenic blocks among the mitochondrial genomes of these four species was not consistent, indicating that the *S. zeylanica* mitochondrial genome has undergone genomic rearrangements relative to its close relatives. This finding corroborates previous research, indicating that plant mitochondrial genomes frequently undergo genomic rearrangements during evolution, potentially contributing to adaptive evolution of the genome. The extremely non-conservative arrangement of mitochondrial genome sequences among these four *Liliales* species likely reflects their adaptive evolution to environmental changes during their evolutionary history. There are some limitations to the present study that should be pointed out. First, the exclusive focus on two *Smilax* species may limit the broader applicability of the conclusions across the genus. Second, potential assembly errors may persist in highly repetitive genomic regions. Third, computationally predicted RNA editing sites remain experimentally unverified. To address these limitations, future research will prioritize expanding taxonomic sampling, refining algorithms for repetitive sequence assembly, and experimentally validating RNA editing events.

## 5. Conclusions

This study presents the first high-quality mitochondrial genomes of *S. glabra* and *S. zeylanica*, two medicinally significant species within the Smilax genus. Our findings reveal their unique multi-branched mitochondrial architectures, conserved core gene sets (24 PCGs), and species-specific variations in non-core genes, tRNA/rRNA content, repetitive elements, and RNA editing sites (exclusively C-to-U conversions). Notably, mitochondrial genome analysis identified chloroplast-derived sequences, suggesting historical organellar DNA transfer events. Synteny comparisons with three other *Liliales* species further highlight extensive genomic rearrangements and non-conserved gene order, reflecting dynamic evolutionary trajectories in plant mitochondrial genomes. These results not only advance the molecular characterization of the Smilax species, but also provide a critical genomic foundation for authenticating traditional medicinal resources, elucidating molecular diversity, and guiding future studies on plant adaptation and evolutionary mechanisms.

## Data Availability

The data that support the findings of this study are available from the corresponding author upon reasonable request.

## Supplementary Materials

The following supporting information can be downloaded at: https://www.mdpi.com/article/10.3390/genes16040450/s1, Table S1. Mitochondrial genome coding genes of *S. zeylanica* and *S. glabra.* Table S2. The relative synonymous codon usage of each amino acid in the mitochondrial genome of *S. zeylanica* and *S. glabra*.
