# Supplementary material for: Assembly and Comparative Analysis of the Complete Mitochondrial Genomes of Smilax glabra and Smilax zeylanica
Source: Genes (Basel). 2025 Apr 14;16(4):450. doi: 10.3390/genes16040450 (PMC12026766; doi:10.3390/genes16040450)
Supplement: Supplementary file 1 [file genes-16-00450-s001.zip › Supplementary Table S2.pdf]

The relative synonymous codon usage of each amino acid in the mitochondrial genome of S. glabra.

Amino	Codon 1<br>RSCU	Codon 2<br>RSCU	Codon 3<br>RSCU	Codon 4<br>RSCU	Codon 5<br>RSCU	Codon 6<br>RSCU
Ala	GCU	GCA	GCC	GCG		
	1.68	0.99	0.87	0.46		
Arg	AGA	CGA	CGU	CGG	AGG	CGC
	1.42	1.28	1.26	0.76	0.68	0.6
Asn	AAU	AAC				
	1.36	0.64				
Asp	GAU	GAC				
	1.41	0.59				
Cys	UGU	UGC				
	1.19	0.81				
End	UAA	UAG	UGA			
	1.85	0.71	0.44			
Gln	CAA	CAG				
	1.55	0.45				
Glu	GAA	GAG				
	1.39	0.61				
Gly	GGA	GGU	GGG	GGC		
	1.44	1.36	0.7	0.5		
His	CAU	CAC				
	1.64	0.36				
Ile	AUU	AUA	AUC			
	1.37	0.84	0.79			
Leu	UUA	CUU	UUG	CUA	CUC	CUG
	1.42	1.27	1.26	0.84	0.64	0.57
Lys	AAA	AAG				
	1.18	0.82				
Met	AUG					
	1.0					
Phe	UUU	UUC				
	1.12	0.88				
Pro	CCU	CCA	CCC	CCG		
	1.42	1.16	0.79	0.63		
Ser	UCU	UCA	AGU	UCC	UCG	AGC
	1.39	1.18	1.05	1.0	0.83	0.55
Thr	ACU	ACC	ACA	ACG		
	1.37	0.99	0.99	0.65		
Trp	UGG					
	1.0					
Tyr	UAU	UAC				

	1.54	0.46				
Val	GUU	GUA	GUG	GUC		
	1.17	1.09	0.97	0.77		

The relative synonymous codon usage of each amino acid in the mitochondrial genome of S. zeylanica.

Amino	Codon 1 RSCU	Codon 2 RSCU	Codon 3 RSCU	Codon 4 RSCU	Codon 5 RSCU	Codon 6 RSCU
Ala	GCU	GCA	GCC	GCG		
	1.6	0.96	0.92	0.52		
Arg	AGA	CGA	CGU	CGG	AGG	CGC
	1.4	1.27	1.21	0.79	0.74	0.6
Asn	AAU	AAC				
	1.32	0.68				
Asp	GAU	GAC				
	1.34	0.66				
Cys	UGU	UGC				
	1.17	0.83				
End	UAA	UAG	UGA			
	1.8	0.77	0.43			
Gln	CAA	CAG				
	1.53	0.47				
Glu	GAA	GAG				
	1.36	0.64				
Gly	GGA	GGU	GGG	GGC		
	1.43	1.34	0.7	0.53		
His	CAU	CAC				
	1.56	0.44				
Ile	AUU	AUA	AUC			
	1.31	0.85	0.84			
Leu	UUA	CUU	UUG	CUA	CUC	CUG
	1.37	1.28	1.23	0.87	0.66	0.59
Lys	AAA	AAG				
	1.14	0.86				
Met	AUG					
	1					
Phe	UUU	UUC				
	1.11	0.89				
Pro	CCU	CCA	CCC	CCG		
	1.37	1.16	0.85	0.62		
Ser	UCU	UCA	AGU	UCC	UCG	AGC
	1.33	1.16	1.03	1.03	0.85	0.6
Thr	ACU	ACC	ACA	ACG		

	1.32	1.05	0.96	0.67
Trp	UGG			
	1			
Tyr	UAU	UAC		
	1.5	0.5		
Val	GUU	GUA	GUG	GUC
	1.15	1.12	0.95	0.78
